# Temporal and sequential changes of glial cells and cytokine expression during neuronal degeneration after transient global ischemia in rats

**DOI:** 10.1186/1742-2094-8-70

**Published:** 2011-06-22

**Authors:** Y Yasuda, T Shimoda, K Uno, N Tateishi, S Furuya, Y Tsuchihashi, Y Kawai, S Naruse, S Fujita

**Affiliations:** 1 Division of Basic Research, Louis Pasteur Center for Medical Research, Kyoto, Japan; 2 Serpha Medicinal Research Institute, Tokyo, Japan; 3 Division of Clinical Research, Louis Pasteur Center for Medical Research, Japan; 4 Division of Clinical Pathology, Louis Pasteur Center for Medical Research, Japan; 5 Department of Medical Informatics, Meiji University of Integrative Medicine, Kyoto, Japan; 6 Department of Radiology, Kyoto Prefectural University of Medicine, Kyoto, Japan

## Abstract

**Background:**

How glial cells and cytokines are associated with the progression of delayed neuronal death induced by transient global ischemia is still unclear. To further clarify this point, we studied morphological changes in glial cells (microglial cells and astrocytes), and cytokine protein levels, during the progression of neuronal cell loss in CA1 (Cornu Ammonis 1) of the hippocampus after transient global ischemia.

**Methods:**

Morphological changes in glial cells were studied immuno-histochemically. Nine cytokines (IL-1α, IL-1β, IL-2, IL-4, IL-6, IL-10, GM-CSF, IFN-γ and TNF-α) were simultaneously measured by a multiplexed bead-based immunoassay from 6 h to day21 after transient four vessel occlusion (4VO) in rats.

**Results:**

During the process of neuronal loss, we observed four distinct phases: (1) lag phase day0-2 (no NeuN+ cell loss observed), (2) exponential phase day2-7 (NeuN+ cells reduced in number exponentially), (3) deceleration phase day7-14 (reduction rate of NeuN+ cells became low), (4) stationary phase day14 onward (NeuN+ cell loss progressed no longer). In the lag phase, activated glial cells were observed in the entire hippocampus but later were gradually restricted to CA1. Cytokine protein levels in the lag and exponential phases were lower than in the deceleration and stationary phases. IL-1α, IL-1β, IL-4, IL-6 and IFN-γ in 4VO were significantly higher in all four phases than in sham. Compared with sham level, GM-CSF was significantly high in the deceleration phase. TNF-α was significantly high in both the deceleration and stationary phases.

**Conclusion:**

Ischemic stress in 4VO activated glial cells in areas beyond CA1 in the lag phase. Pyramidal neurons were injured in CA1 from the end of the lag phase and then neuronal cells reduced in CA1 in the exponential phase. After neuronal death began, the influence of dead cells on glial cells and cytokine expression gradually became stronger than the influence by ischemic stress. Therefore, from the deceleration phase, changes in glial cells and cytokine production were likely caused by dead cells. Cytokine interaction in the microenvironment may determine the functions of IL-1α, IL-1β, IL-4, IL-6 and IFN-γ in all four phases. The function of GM-CSF and TNF-α in the deceleration phase may be neurotrophic.

## Background

Transient brain ischemia is known to cause delayed neuronal death, resulting in an expansion of the injured area after recirculation. Transient global ischemia model is generally used to analyze the mechanism of neuronal cell death caused by transient brain ischemia, because transient global ischemia induces delayed pyramidal neuronal cell death only in CA1 of the hippocampus on day2 or 3 after recirculation [1-3]. Transient global ischemia activates microglial cells and astrocytes, and up-regulates the production of inflammatory cytokine. Activated microglial cells and astrocytes play an important role in the progression of ischemic injury by producing cytokines [4]. Suppressing microglial cell activation protects against neuronal death induced by transient global ischemia [5].

An increase in inflammatory cytokines, such as IL-1β, IL-6 and TNF-α at early time points after transient global ischemia has been reported [6-11]. Antibodies that neutralize IL-1β or TNF-α, the soluble form of IL-1β or TNF-α receptor, and IL-1β analogue all function to reduce the injury caused by brain ischemia in rodents [12-16]. These reports suggest that IL-1β and TNF-α trigger neuronal death in CA1 and that inflammatory cytokines are closely associated with neuronal degeneration in ischemic injury [9,17-20]. Brain born- as well as peripheral-born cytokines contribute to ischemic injury progression and repair [21,22].

Inflammatory cytokines are individually pleiotrophic, and vary in pleiotrophic aspects in combination with other cytokines. Since cytokine interaction affects cytokine function, it is necessary to study multiple cytokines simultaneously in order to understand their role in the progression of ischemic injury. Cytokine production is generally measured through mRNA levels [23]. However, since newly synthesized cytokine mRNA is not always transcribed to produce protein, it is more suitable to simultaneously measure the changes in protein levels of multiple cytokines for an accurate understanding of cytokine function [24,25].

Knowledge of the pattern of multiple cytokine expression in the hippocampus is required to understand the inflammation associated with neuronal death induced by transient global ischemia. However to date, there are no known reports about the profile of multiple cytokine protein levels in the hippocampus.

The aim of this study is to reveal how glial cells and cytokines are related to neuronal cell degeneration induced by transient global ischemia in rats. A global ischemic model was prepared by a 10 min four-vessel occlusion (4VO) followed by re-circulation in rats. After recirculation, the changes in glial cells and cytokine expression during neuronal reduction in CA1 were studied by immuno-histochemical methods and multiplexed bead-based immunoassay, respectively. A total of nine cytokines, IL-1α (interleukin-1α), IL-1β, IL-2, IL-4, IL-6, IL-10, GM-CSF (granulocyte-macrophage colony stimulating factor), IFN-γ (interferon-γ) and TNF-α (tumor necrosis factor-α), were simultaneously measured in this study.

## Materials and methods

### Animals and surgical procedures

Male Wistar rats (Cr1j:WI, 7 weeks old) obtained from Charles River Japan Inc. (Hamamatsu, Japan) were housed in a temperature controlled room for seven days prior to surgery. All experiments were conducted in accordance with the Guidelines for Animal Experiment compiled by the Animal Care and Animal Use Ethical Committee at the Louis Pasteur Center for Medical Research.

**Figure 1:**
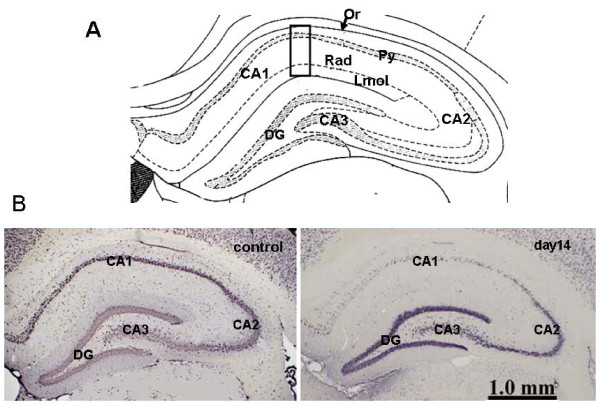
**Neuronal cell death only in CA1 of hippocampus**. A shows the structure of the hippocampus. CA1-3: Cornu Ammonis 1-3, Or: oriens layer of CA1, Py: pyramidal cell layer of CA1, Rad: radiatum layer of CA1, Lmol: lacunosum-molecular layer of CA1, GD: dentate gyrus. B shows microscopic images of the hippocampus immunostained with an anti-NeuN antibody. On day14, NeuN+ cell reduction was observed in CA1 but not in CA2-3.

Rats were subjected to transient global ischemia by clamping the carotid arteries bilaterally utilizing a combination of methods described in previous reports [1,2,26]. In brief, 24 hours before inducing ischemia, the rats were anesthetized with pentobarbital sodium and the bilateral vertebral arteries were heat-cauterized at each alar foramen using a soldering iron. Both common carotid arteries were then gently isolated and a silicone tube placed around each vessel. The following day, both common carotid arteries were occluded with vascular clamps for 10 min. In sham groups, the same operation was performed but without occlusion of both carotid arteries. Only rats that had lost their righting reflexes during the period of ischemia were used for this study. The changes in glial cells and cytokines were studied on day0.25 (6 hr), 1, 2, 3, 5, 7, 10, 14 and 21 post re-circulation. In this rat model, only CA1 pyramidal neurons died (Figure 1).

### Immunohistochemistry

The rats were anesthetized with diethyl ether and perfused with saline and then perfusion-fixed with 4% paraformaldehyde in 0.1 M phosphate-buffer (PB), pH 7.4. After perfusion-fixation, the brain was dissected and post-fixed for one day in the same fixative. Subsequently, the tissue blocks were embedded in O.C.T. medium (Sakura Finetech Japan Co., Ltd, Tokyo, Japan) and were frozen at -80°C. Coronal 14 μm thick sections were sequentially cut in a cryostat.

The sections were immersed in Block-One (Nacalai Tesque Inc., Kyoto, Japan) for 30 min in order to block non-specific binding, and were incubated with the primary antibodies at 4°C overnight. As primary antibodies, anti-neuronal nuclei (NeuN) antibody (I:200, Millipore Ltd., Billerica, MA, USA), anti-ionized calcium binding adaptor molecule 1 (Iba1) antibody (1:200, Wako Pure Chemical Industries Ltd., Osaka, Japan), and anti-glial fibrillary acidic protein **(**GFAP) antibody (Progen Biotechnik Gmbh, Heiderberg, GM) were prepared to detect neuronal nuclei, microglial cells/macrophages, and astrocytes, respectively. ABC Elite kit (Vector Laboratories Inc., Burlingame, CA, USA) as signal enhancer, and chromogen solution including di-aminobenzidine (DAB, Wako Pure Chemical Industries, Ltd.) and ammonium nickel(II) sulfate hexahydrate (Nacalai Tesque Inc.) [27] were used for the conventional microscopic observation.

The primary area for histological observation in the hippocampus was CA1 which consists of oriens layer (Or), pyramidal cell layer (Py), radiatum layer (Rad) and lacunosum-molecular layer (Lmol) (Figure 1).

### Protein preparation for cytokine measurements

Rats were decapitated under diethyl ether anesthesia and the brains were rapidly removed and dissected into 2 mm thick coronal slices. A section of the hippocampus was removed from the coronal slice and rapidly frozen in dry ice and kept at -80°C.

In preparation for extracting the protein from the hippocampus slices, 100 μl cell lysis buffer (Bio-Plex Cell Lysis Kit, Bio-Rad Laboratories Inc., Hercules, CA, USA) containing protease inhibitor (Sigma P8340, Sigma Ardlich Co., St. Louis, MO, USA) was added to the samples. The samples were sonicated on ice using Handy Sonic UR-20P (Tomy Seiki Co. Ltd., Tokyo, Japan) and centrifuged at 14,000 × g for 5 min at 4°C, the supernatant were then transferred to a tube. Using the Bradford method (Protein Assay Kit, Bio-Rad Laboratories Inc.), we determined the protein concentration of the supernatant and adjusted it to 500 μg/ml using cell lysis buffer for immunoassay.

Cytokines were measured using a multiplexed bead-based immunoassay kit, Bio-Plex rat 9 plex A panel (Bio-Rad Laboratories Inc.), in conjunction with a Cytokine Reagent kit (Bio-Rad Laboratories Inc.) and a Bio-Plex Protein Array System (Bio-Rad Laboratories Inc.). The 9 Plex A panel consisted of the following analytes: IL-1α, IL-1β, IL-2, IL-4, IL-6, IL-10, GM-CSF, IFN-γ and TNF-α. Results were analyzed using Bio-Plex Manager 30 software (Bio-Rad Laboratories Inc.). The reliability and accuracy of the Bio-Plex rat cytokine assay kit is supported by prior reports [28].

### Statistical analysis

For statistical analysis, half the value of the lowest limit was used as a substitute for values that fell out of range (below the lowest limit). Data are presented as mean ± SEM. Cytokine changes during the process of neuronal cell degeneration in CA1 were analyzed by ANOVA followed by the Dunnett method. Differences between 4VO and sham groups at each time point were analyzed with an unpaired *t *test. P values of 0.05 or less were considered statistically significant.

## Results

### Reduction of CA1 pyramidal neurons

**Figure 2:**
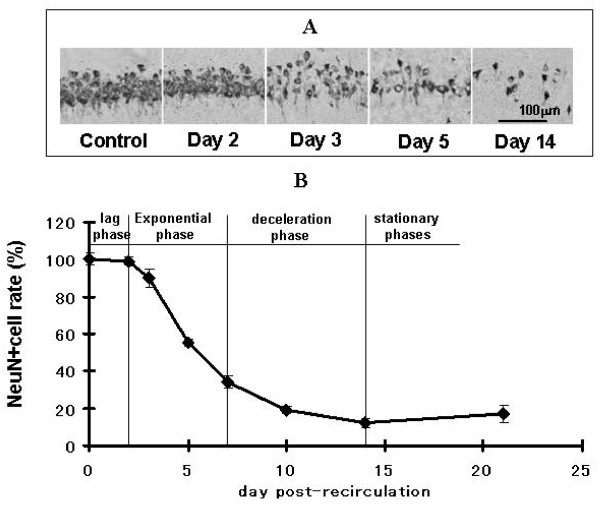
**Reduction of NeuN+ cells after recirculation**. A are microscopic images of a part of CA1 Py, indicated by the rectangle in Figure 1A, immunostained with an anti-NeuN antibody. On day3, NeuN+ cell reduction was observed. This reduction continued from day5 to day14. Graph B shows the reduction curve of NeuN+ cells in CA1 Py. In this graph, NeuN+ cell percentage is calculated by dividing the no. of NeuN+ cell in 4VO by the no. of NeuN+ cell in the normal rats in an area of the same size. Each data point shows the mean ± SEM of results from 5 rats. The amount of NeuN+ cells in CA1 on day3, 5, 7, 14 and 21 were 90 ± 5.1, 55 ± 2.1, 34 ± 3.5, 12 ± 2.3% and 17 ± 5%, respectively. The process of neuronal reduction after recirculation was divided into 4 phases: lag, exponential, deceleration and stationary phases.

In this global model, neuronal damage was visible only in CA1 of the hippocampus and no other region. The reduction in the number of CA1 pyramidal neurons after global ischemia was studied by immunostaining with anti-NeuN antibody and then NeuN+ cells were counted (Figure 2). The number of NeuN+ cells in CA1 on day2 was slightly different from that in normal rats. Even though NeuN+ cells reduced on day3, the number of surviving cells was not statistically different from normal rats, but a statistically significant reduction was observed on day5. In the sham group, no neuronal reduction was observed.

These results reveal that the process of neuronal reduction can be divided into four phases: (1) lag phase when very little NeuN+ cell loss was observed (day0-2), (2) exponential phase when NeuN+ cells reduced exponentially (day2-7), (3) deceleration phase when the rate of reduction became lower (day7-14), (4) stationary phase when additional neuronal loss was no longer observed (after day14).

### Activation of microglial cells

**Figure 3:**
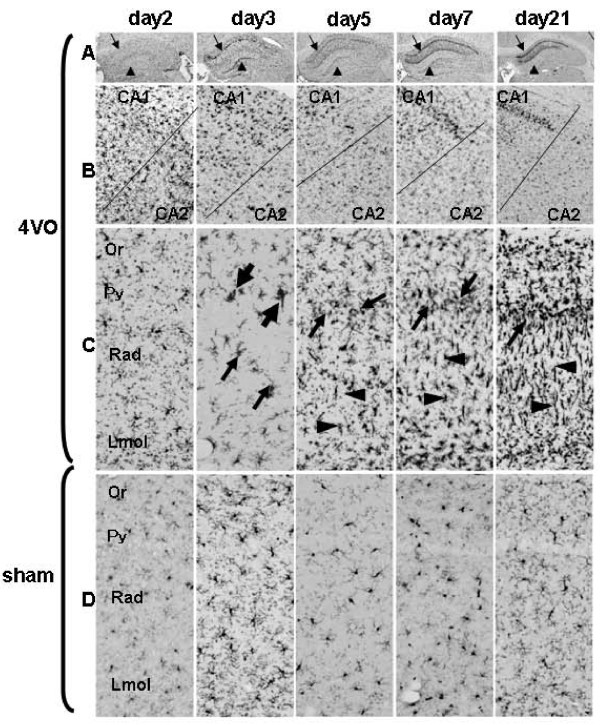
**Change of microglial cells after recirculation**. Anti-Iba1 antibody was used to immunostain microglial cells. Rows A, B, C, and D represent different sections of the hippocampus. A: the entire hippocampus in 4VO, B: border between CA1 and CA2 in 4VO, C: larger magnification of CA1 of the hippocampus in 4VO, D: larger magnification of CA1 of the hippocampus in sham. C and D were taken from the rectangular area shown in Figure 1A. Arrows and arrow heads in row A show Py and dentate gyrus, respectively. In row B, the upper and lower sides of the images show CA1 and CA2, respectively. Arrows and arrowheads in row C indicate amoeboid microglial cells and rod cells, respectively. On day2, microglial cells began to be hypertrophic in the entire hippocampus. On day3, amoeboid cells (arrow) were observed in CA1 and clusters including 2 or 3 microglial cells (large arrows) were around Py. Between day5 and 21, Iba1+ amoeboid cells (arrow) in Py, rod cells with short processes in Rad (arrow head) and hypertrophic cells in Or and Lmol of CA1 in the hippocampus were observed.

After recirculation, microglial cell activation was immuno-histochemically studied using anti-Iba1 antibody (Figure 3). Day2 post-recirculation, Iba1+ cells in the entire hippocampus were slightly hypertrophic compared with sham samples. On day3, Iba1+ large amoeboid cells were observed in CA1 and dentate gyrus (Figure 3). Iba1+ clusters consisting of 2 or 3 cells were observed around Py on day3. Apart from on day3, Iba1+ clusters were not observed at other time points. On day5 post-recirculation, Iba1+ cells increased especially in Rad, additionally Iba1+ small amoeboid cells with weak immunostaining intensity in Py were also observed. On day7, the shape of Iba1+ hypertrophic cells varied among layers: Iba1+ thin-rod cells with short processes were observed in Rad, small amoeboid cells in Py and hypertrophic cells with many processes in Or and Lmol. These morphological characteristics in CA1 not only remained but were accentuated on day21. Our finding agrees with Sugawara et al. [29] who reported the appearance of rod cells after transient global ischemia. In sham-operated rats, the morphological change of Iba1+ cells was slight, but clearly recognizable from the immunostaining intensity and morphological change of their processes.

These results mean that microglial cell morphology is affected even by sham operation that stops blood flow in the bilateral vertebral arteries only. These finding also suggests that in the lag phase, microglial cells are activated in the entire hippocampus. After neuronal cell reduction began, the distribution of activated microglial cells was gradually restricted to CA1 of the hippocampus.

### Activation of astrocytes

**Figure 4:**
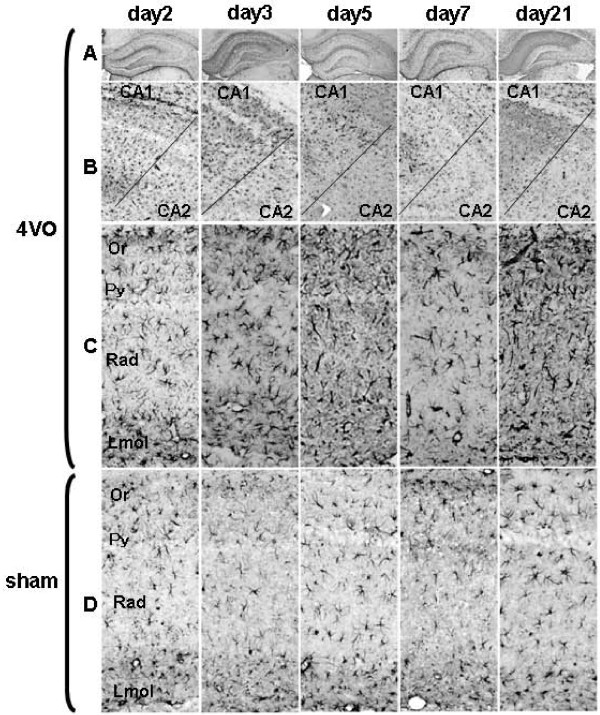
**Morphological change of astrocytes after recirculation**. Astrocytes were detected by immunostaining for GFAP. Each image represents one time point. Rows A, B, C, and D represent a different section of the hippocampus. A: the entire hippocampus in 4VO, B: border between CA1 and CA2 in 4VO, C: a part of CA1 of the hippocampus in 4VO, D: a part of CA1 of the hippocampus in sham. C and D were taken from the rectangular area shown in Figure 1A. In row B, the upper and lower sides of the images show CA1 and CA2, respectively. On day2, astrocytes were more hypertrophic (C) than in sham (D). From day3 to day21, astrocytes in CA1 were more hypertrophic than those in CA2 of the hippocampus.

The activation of astrocytes was studied by immunostaining for GFAP (Figure 4). In 4VO on day2, GFAP+ cells in the entire hippocampus were more hypertrophic than in the sham samples. Astrocytes were also more hypertrophic in CA1 than in CA2 from day3 to 21. The immunostaining intensity increased on day7 and 21. In sham, no remarkable morphological changes were observed except slight changes in CA1. These results show that activated astrocytes were distributed in the entire hippocampus in the lag phase, but were gradually restricted to CA1 in later phases.

### Changes in cytokine protein levels in the hippocampus

**Figure 5:**
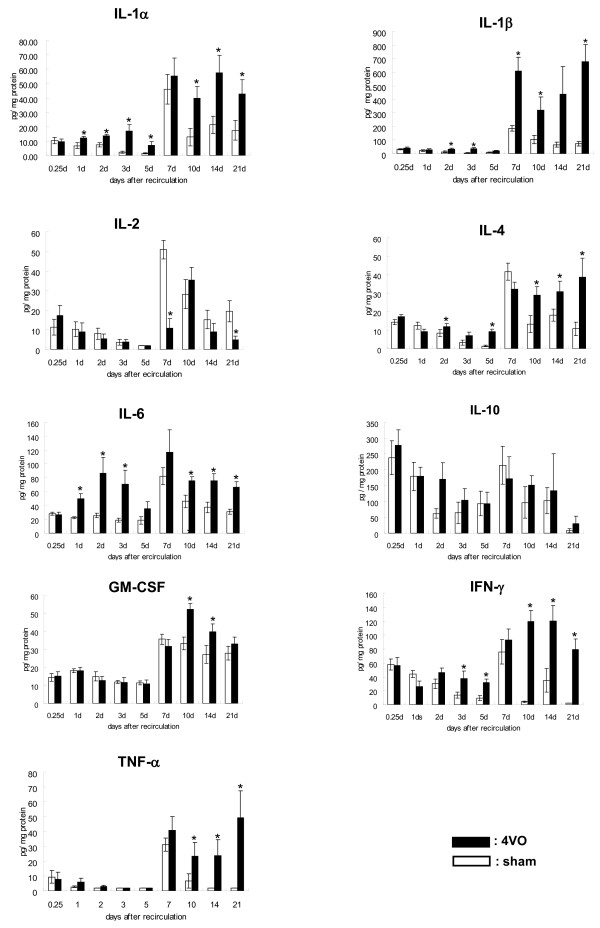
**The change of cytokine protein level in hippocampus after recirculation**. All protein levels were simultaneously measured using a multiplexed bead-based immunoassay. Each data point indicates the mean ± S.E.M. (sham = 6, 4VO = 6-8). *p < 0.05, significantly different from sham.

IL-1α, IL-1β, IL-2, IL-4, IL-6, IL-10, GM-CSF, IFN-γ and TNF-α in the hippocampus post-recirculation were simultaneously measured in 4VO and sham from 6 h (day 0.25) until day21, and the changes were analyzed by ANOVA (Figure 5).

Cytokine expression was affected by both 4VO and sham operation (Figure 5). IL-1α, IL-1β, IL-2, IL-4, IL-6, GM-CSF, IFN-γ and TNF-α protein levels were significantly higher on day7, compared with their levels on day5. In 4VO, IL-1α, IL-1β, GM-CSF, IFN-γ and TNF-α high protein levels remained until day21. IL-2 protein level decreased from 6 h to day5, increased from day7 until day10 and decreased again from day14. IL-6 protein level peaked on day2, decreased until day5, increased again on day7 and remained steady until day21. In sham, even though the protein levels of IL-1α, IL-1β, IL-2, IL-4, IL-6, IFN-γ and TNF-α increased on day7, they decreased on day10, and afterward, these protein levels did not change significantly. No significant difference in IL-10 protein level was observed between day5 and 7.

The difference in protein level between 4VO and sham at each time point was analyzed using student t-test (Table 1). At 6 h IL-1α, IL-1β, IL-2, IL-4, IL-6, GM-CSF, IFN-γ and TNF-α protein levels did not differ significantly between 4VO and sham. On day1 however, IL-1α and IL-6 levels were significantly higher (p < 0.05) in 4VO than in sham. On day2, IL-1β and IL-4 levels, in addition to IL-1α, and IL-6, were significantly higher than in sham (p < 0.05). Afterward IL-1α, IL-1β, IL-4, IL-6 and IFN-γ levels were high (0.05 < p < 0.1) or significantly high (p < 0.05) until day5. On day7, only IL-1β level was significantly higher (p < 0.05) and IL-2 level significantly lower (p < 0.05) than sham. From day10 to 14, IL-1α, IL-1β, IL-4, IL-6, GM-CSF, IFN-γ and TNF-α levels were high (0.05 < p < 0.1) or significantly high (p < 0.05), compared with their sham levels. On day21, IL-1α, IL-1β, IL-4, IL-6, IFN-γ and TNF-α levels in 4VO were significantly higher than in sham and GM-CSF decreased to the sham level. IL-2 protein level at each time point in 4VO was similar to, or lower than the sham level. IL-10 protein level at each time point was not significantly different between 4VO and sham.

**Table 1:** Cytokines expressed significantly higher or lower than sham in each phase of neuronal reduction. P < 0.05 by t-test

	~lag phase		exponential phase			deceleration phase		stationary phase~
**day after recirculation**	**day1**	**day2**	**day3**	**day5**	**day7**	**day10**	**day14**	**day21**

	IL1α, IL-6	IL-1α, IL-1β	IL-1α, IL-1β	IL-1α, IL-4	IL-1β	IL-1α, IL-1β	IL-1α, IL-4	IL-1α, IL-1β
**higher**		IL-4, IL-6	IL-6, IFN-γ	IFN-γ		IL-4, IL-6	IL-6,GM-CSF	IL-4, IL-6
						GM-CSF,	IFN-γ, TNF-α	IFN-γ,TNF-α
						TNF-α		
**lower**	none	none	none	none	IL-2	none	none	IL-2

## Discussion

In this experiment, the process of neuronal death caused by transient global ischemia was analyzed, focusing on the prolonged changes in glial cells and multiple cytokine expression (IL-1α, IL-1β, IL-2, IL-4, IL-6, IL-10, GM-CSF, IFN-γ and TNF-α). The reduction curve of neuronal cells in CA1 after 4VO (Figure 3) was S shaped. The process of neuronal cell degeneration was divided into four phases: lag, exponential, deceleration and stationary phases. We found that the changes of glial cell morphology and cytokine expression differed among these four phases of neuronal reduction.

### Glial cell activation after 4VO

In this 4VO experiment, activated microglial cells and astrocytes were observed in the entire hippocampus in the lag phase. After neuronal reduction was observed, both microglial cells and astrocytes gradually became more hypertrophic in CA1 than CA2-3. These results suggest that transient global ischemia activates microglial cells and astrocytes in an area wider than CA1 of the hippocampus in the lag phase but after the exponential phase, the activation of both glial cells was gradually restricted mainly to CA1. Glial cells in CA1 also increased in the deceleration phase.

Iba1+ clusters around Py on day3 consisted of activated microglial cells in the prophase or metaphase of the mitosis (see additional file 1). Fujita et al. [30] prior reported that microglial cells actively proliferated in the initial stage of brain injury supports our findings. The existence of Iba1+ clusters on day3 indicates that the activated microglial cells, which may have migrated around Py or had already existed there, actively proliferated and Iba1+ clusters were created.

In order to shed more light on the relationship between neuronal death and NeuN+ cell loss, we studied DNA fragmentation using terminal deoxynucleotidyl transferase dUTP nick end labeling (TUNEL) staining. The result from this staining showed that neuronal DNA damage had already started on day2 (see additional file 2). There was a time lag between when neuronal death was detected by TUNEL and the occurrence of NeuN+ cell loss.

Our results, combined with those from previous reports [29], suggest that glial cell activation is mainly a result of transient global ischemia in the lag phase. Neuronal cell death gradually evoked glial cell activation in the exponential phase and then dead cells stimulated the proliferation of glial cells.

### Cytokine expression after 4VO

In previous experiments using the rat model, an increase in IL-1β and IL-6 mRNA levels after recirculation within hours after transient global ischemia was reported [6,8,31,32]. In our experiment, we did not observe an increase in inflammatory cytokine protein levels at 6 h, but our immuno-histochemical study showed that in the hippocampus, IL-1β immuno-intensity in astrocytes was higher in 4VO than in sham (see additional file 3). These results point to the possibility that IL-1β is related to neuronal damage induced by 4VO. On day1, an increase in IL-1α and IL-6 was detected by bead-based immunoassay. The beads assay and immuno-histochemical study delivered different results, maybe due to the fact that cytokine in the soluble form was reduced during brain fixation and immunostaining process. A previous report using gerbil model showed that TNF-α protein level increased at 1 h, decreased to the control level and increased again on day1 [7]. It was further reported that IL-1β and IL-6 protein levels increased after 6 h and 3 h respectively, decreased and then increased again on day1. The disparity between Saito et al's results [7] and those of this study may be due to the difference in the animal model or breeder that was used as well as the fact that we used a bead-based assay while Saito et al. employed an enzyme immunoassay. An increase of IL-1β and TNF-α in the early phase after re-circulation is thought to trigger a signal transduction towards neuronal cell death in CA1. In our rat model, IL-1β, IL-6 and TNF-α protein levels may have increased earlier than 6 h after re-circulation and then decreased to the sham level at 6 h.

The protein levels of IL-1α, IL-1β, IL-2, IL-4, IL-6, GM-CSF, IFN-γ and TNF-α were up-regulated on day7 in both 4VO and sham, but protein levels in other cytokines except IL-1β and IL-2 were not significantly different between 4VO and sham. On the same day, all glial cells in CA1 were strongly activated in 4VO but in sham there was only a slight morphological change of glial cells. We observed also that more microglial cells in 4VO on day7 was more activated than on day5. Additionally, our immuno-histochemical study of IL-1β distribution in the hippocampus showed that IL-1β staining intensity in activated astrocytes in 4VO was stronger than sham on day7 (see additional file 3). In 4VO, activated astrocytes and microglial cells in the hippocampus might be involved in the increase in cytokine levels on day 7. In sham, these results suggest that sham operation (heat-cauterization of alar foramen) may affect cytokine protein levels in the hippocampus without any pronounced activation of microglial cells and astrocytes. These results may also indicate that in addition to cytokines produced in glial cells, peripheral-born cytokines and/or brain-born cytokines produced outside of the hippocampus can contribute to the increase in cytokine protein levels on day7 and cytokine producing cells might be different between 4VO and sham. On day7, only IL-1β was significantly higher in 4VO than in sham and IL-2 in sham was significantly higher than in 4VO. The change in cytokine protein levels after day10 was different in 4VO and sham. In 4VO, IL-1α, IL-1β, IL-4, IL-6, GM-CSF, IFN-γ and TNF-α protein levels were higher or significantly higher than in sham and these cytokines maintained a high protein level during the deceleration phase. In sham, on the other hand, most cytokines were up-regulated on day7 and then decreased on day10. It is possible that day7 may be an important point in 4VO and sham when the continuation or end of inflammation is decided. IL-2 is a neuro-regulatory cytokine in the brain [33] and IL-1β has both neurotrophic and neurotoxic functions. IL-1β in 4VO and IL-2 in sham on day7 may be involved in regulating other cytokine expression after day7.

In the exponential and deceleration phases the protein levels of IL-1α, IL-1β, IL-4, IL-6 and IFN-γ were significantly higher in 4VO than in sham, thereby making it difficult to ascertain how these cytokines contribute to the process of neuronal loss. IL-1α, IL-1β, IL-6 and TNF-α have both neurotoxic and neurotrophic functions. The role of each cytokine in the progression of neuronal cell death or repair might be revealed through cytokine interaction. It could be that the interaction among cytokines with protein levels that were significantly higher than sham in the lag and exponential phases might lead to the progression of neuronal cell death. In the deceleration and early stationary phases after day7, glial cells in CA1 of the hippocampus remained activated in 4VO but were not activated in sham. In the deceleration and early stationary phases after day7, the interaction among cytokines with protein levels that were significantly higher in 4VO than in sham, may have led to the end of neuronal death, and the cleaning of dead cell debris mainly with the help of activated microglial cells/macrophages. However, more studies are required to fully understand this relationship. In addition to understanding the profile of multiple cytokines after re-circulation, we believe that knowing when neuronal death is triggered may also be important for developing a wider therapeutic window.

Significantly higher protein level in GM-CSF was observed only in the deceleration phase in 4VO, compared with sham. GM-CSF counteracts apoptosis, is neurotrophic and stimulates the differentiation of granulocyte/macrophage [34]. GM-CSF administration suppresses delayed neuronal cell death after focal ischemia [35]. An increase in GM-CSF only in the deceleration phase suggests that GM-CSF may help to protect neuronal cells from apoptosis and repair the injury induced by transient global ischemia.

No significant difference between 4VO and sham in IL-10, an anti-inflammatory cytokine, was observed after transient global ischemia in our experiment but it is reported that IL-10 administration suppresses neuronal cell reduction caused by ischemia [36].

Suppressing the function of IL-1α, IL-1β and TNF-α [12-16], anti-inflammatory cytokines such as IL-10, and delayed neuronal cell death suppressors such as GM-CSF, are effective for suppressing delayed neuronal cell death after transient global ischemia. Detailed information of the function of each cytokine in the lag, exponential and deceleration phases might be helpful in developing methods for mitigating ischemic insult.

More information about the relationship between neuronal death and cytokine profile can lead to the development of new cytokine therapy based on the changes in cytokine profile and the progression time of neuronal death after transient global ischemia.

## Conclusions

The changes in glial cells and cytokine expression were characteristic in each phase of neuronal cell degeneration. In the lag phase, glial cells and cytokine expression were mainly affected by ischemic stress, and from the exponential phase dead cells gradually influenced the change in glial cells and cytokine expression. The function of IL-1α, IL-1β, IL-4, IL-6 and IFN-γ may be decided by cytokine balance in the microenvironment. In the lag and exponential phases, cytokine balance may shift to being more neurotoxic and in the deceleration and stationary phase to being more neurotrophic.

## List of abbreviations used

CA: Cornu Ammonis in hippocampus; DAB: di-aminobenzidine; DG: dentate gyrus; GFAP: glial fibrillary acidic protein; GM-CSF: granulocyte-macrophage colony stimulating factor; Iba1: ionized calcium binding adaptor molecule 1; IFN-γ: interferon-γ; IL: interleukin; Lmol: lacunosum-molecular layer in CA1; NeuN: neuronal nuclei; Or: oriens in CA1; Py: pyramidal cell layer in CA1; Rad: radiatum layer in CA1; TNF-α: tumor necrosis factor-α; TUNEL: terminal deoxynucleotidyl transferase dUTP nick end labeling

## Competing interests

The authors declare that they have no competing interests.

## Authors' contributions

YY carried out the preparation for morphological observation and contributed to drafting of the manuscript. TS and KU prepared the protein extract and measured the cytokine protein levels. NT prepared the transient global ischemic rat model. ^3^SF, YK and SN helped with statistical analysis. YT did microscopic observation. ^1^SF participated in the design of the studies and helped to draft the manuscript. All authors read and approved the final manuscript.

## Supplementary Material

Additional file 1 **Mitosis of microglial cells after recirculation**. Presence of activated microglial cells was detected by immunostaining for Iba1 and DNA was stained with propidium iodide (PI) after treatment with RNase. Red and green indicate DNA and Iba1, respectively. Activated microglial cells in meta- (arrow) and pro - (arrow head) phases were observed in CA1. Click here for file

Additional file 2 **TUNEL positive pyramidal neuron in CA1 after re-circulation**. Fragmented DNA was detected, using DeadEndTM Colorimetric TUNEL System (Promega Co., Wisconsin, USA). Methyl green was used to counter-stain. Arrows show TUNEL+ neuronal cells. TUNEL+ neuronal cells were observed on day2 after re-circulation. Click here for file

Additional file 3 **Immunostaining images for GFAP and IL1β at 6 h and on day7 after re-circulation**. Red and green indicate GFAP and IL-1β, respectively. IL-1β was distributed in GFAP+ cells. Staining intensity in 4VO was stronger than in sham. Click here for file
