# Supplementary figures and images for: Temporal and sequential changes of glial cells and cytokine expression during neuronal degeneration after transient global ischemia in rats
Source: J Neuroinflammation. 2011 Jun 22;8:70. doi: 10.1186/1742-2094-8-70 (PMC3131233; doi:10.1186/1742-2094-8-70)

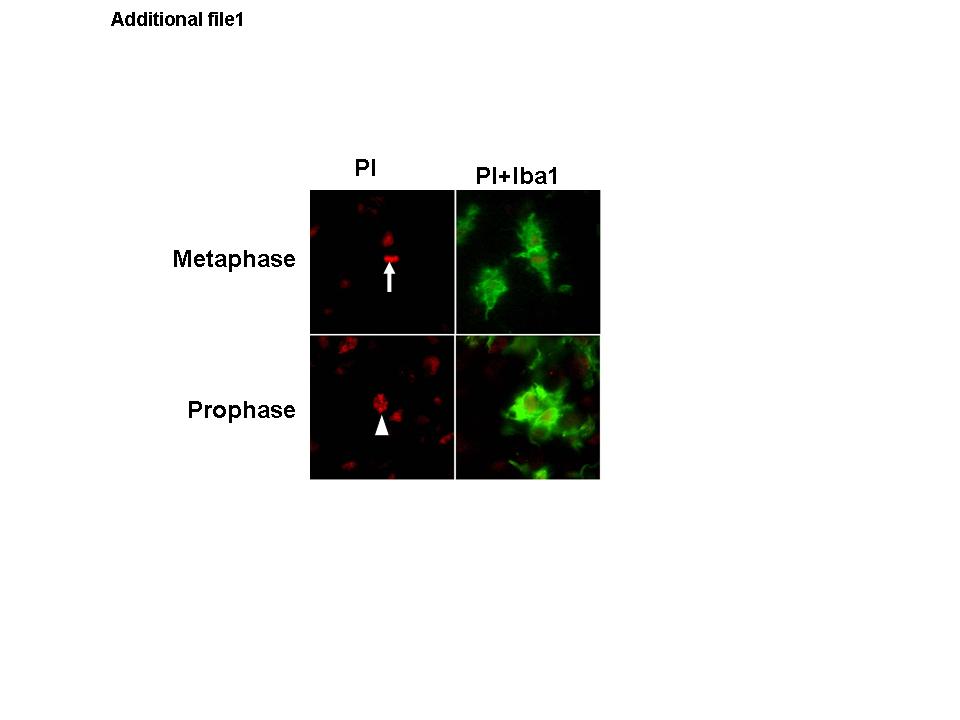

Supplement: Additional file 1 — Mitosis of microglial cells after recirculation. Presence of activated microglial cells was detected by immunostaining for Iba1 and DNA was stained with propidium iodide (PI) after treatment with RNase. Red and green indicate DNA and Iba1, respectively. Activated microglial cells in meta- (arrow) and pro - (arrow head) phases were observed in CA1. [file 1742-2094-8-70-S1.JPEG]

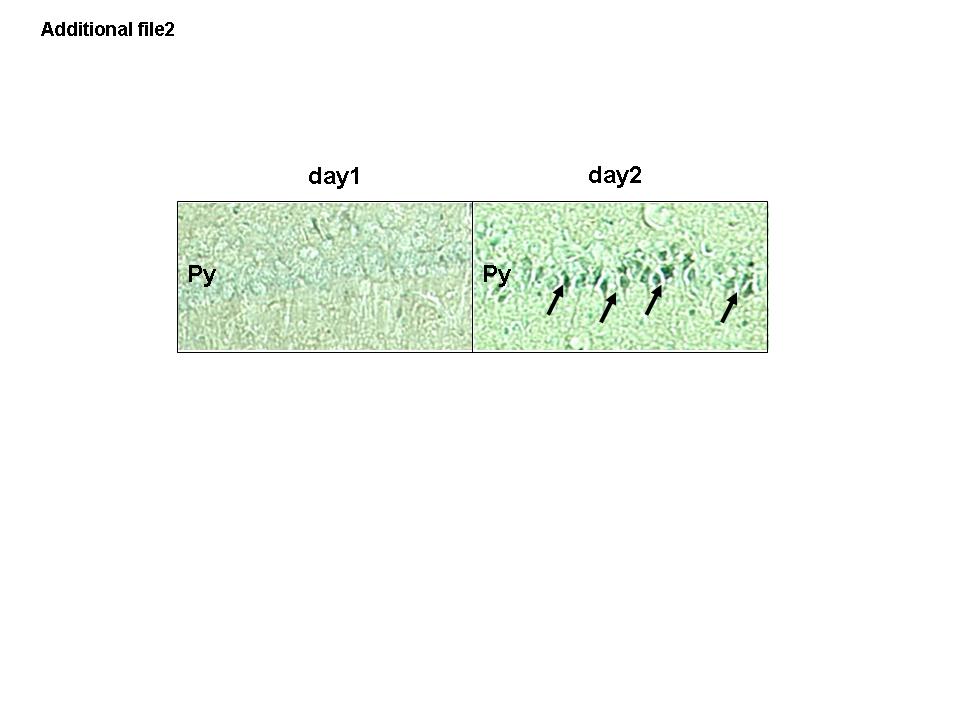

Supplement: Additional file 2 — TUNEL positive pyramidal neuron in CA1 after re-circulation. Fragmented DNA was detected, using DeadEndTM Colorimetric TUNEL System (Promega Co., Wisconsin, USA). Methyl green was used to counter-stain. Arrows show TUNEL+ neuronal cells. TUNEL+ neuronal cells were observed on day2 after re-circulation. [file 1742-2094-8-70-S2.JPEG]

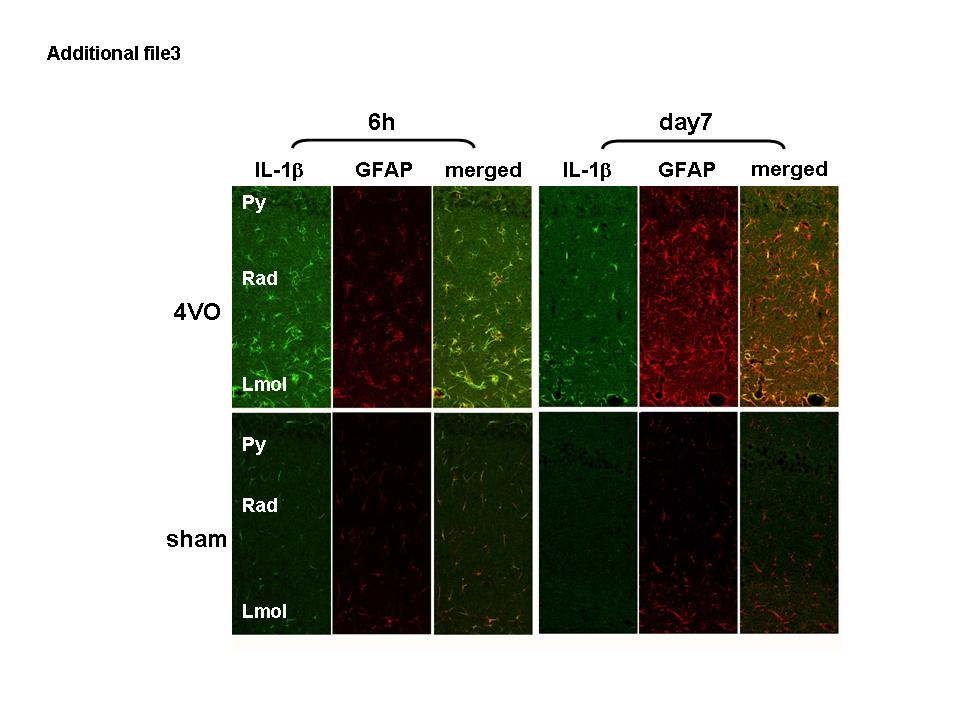

Supplement: Additional file 3 — Immunostaining images for GFAP and IL1β at 6 h and on day7 after re-circulation. Red and green indicate GFAP and IL-1β, respectively. IL-1β was distributed in GFAP+ cells. Staining intensity in 4VO was stronger than in sham. [file 1742-2094-8-70-S3.JPEG]
